# ECG interpretation abilities in clinical practice: Examining the role of expertise, age, and gender

**DOI:** 10.1097/MD.0000000000042401

**Published:** 2025-08-08

**Authors:** Eyad Talal Attar

**Affiliations:** a Department of Electrical and Computer Engineering, King Abdulaziz University, Jeddah, Saudi Arabia.

**Keywords:** age differences, arrhythmia detection, cardiovascular abnormalities, clinical experience, cognitive load, diagnostic accuracy, ECG training, electrocardiogram (ECG) interpretation, eye-tracking technology, Gaze patterns, gender differences, healthcare professionals, medical education

## Abstract

This study investigated the competence of health professionals at all levels of expertise, medical students, residents, and cardiology consultants, in diagnosing electrocardiography (ECG) abnormalities, with the primary emphasis on professional experience, gender, age, and years in practice. ECG remains a foundational method to diagnose cardiac functions; however, there are disparities among healthcare providers’ interpretation skills. Typically, previous studies have examined narrow cohorts, which limits insights into ECG interpretative abilities across the healthcare spectrum. In this study, 72 participants completed a comprehensive ECG interpretation assessment involving common and complex abnormalities, while cognitive engagement was tracked via eye-tracking metrics to evaluate interpretation processes. Data were collected from the professionals using surveys and eye-tracking technology to evaluate ECG interpretation skills. Informed consent was obtained from the participants, and the Qatar Biomedical Research Institute’s IRB: QBRI-IRB-2020-01-009 approved the ethical standards followed. The results showed significant differences in diagnostic accuracy based upon expertise. Cardiology consultants showed the highest accuracy, while younger participants between 26 and 30 years outperformed the older groups, and males generally had higher recognition rates overall compared to females. The eye-tracking analysis highlighted prolonged engagement with complex ECG segments, which suggested interpretative challenges. Importantly, targeted educational interventions improved participants’ recognition accuracy notably. These findings underscore tailored training programs’ potential to enhance ECG interpretation skills, and ultimately improve diagnostic accuracy and patient outcomes. Further, the Random Forest model achieved 81% accuracy in classifying long versus short ECG readings. This study highlights critical gaps in ECG proficiency and advocates for continuous, demographics-based training enhancements. Future research should prioritize longitudinal studies to evaluate targeted training interventions’ effect on enhancing ECG interpretation skills across a diverse range of healthcare professionals.

## 1. Introduction

Arrhythmias, ischemic heart diseases, and all disorders of cardiac function are among the many cardiac illnesses that can be diagnosed with electrocardiography (ECG).^[1]^ ECG interpretation proficiency is crucial for appropriate intervention and better results.^[2]^ However, depending on influencing factors like education, age, sex, clinical experience, and expertise, doctors’ abilities to identify abnormal ECGs differ greatly.^[3]^ Recent studies have shown notable differences in the ability of various demographic groups to interpret ECGs, which are impacted by variables like age, gender, and experience level.^[4]^ For example, one study found that younger clinicians were more accurate than their older counterparts at interpreting ECGs, possibly as a result of their greater familiarity with modern training techniques and technology.^[5]^ In addition, another study discovered that older healthcare professionals had trouble identifying subtle ECG abnormalities, which could be a sign of a gradual decline in diagnostic accuracy, even with their extensive experience.^[6]^ Furthermore, recent research has also brought attention to gender differences in ECG interpretation. Concerns regarding possible biases in training and evaluation procedures are raised by a study that revealed female clinicians performed worse on ECG interpretation tests than male clinicians.^[7]^ These conflicting findings emphasize the need for specialized educational interventions and the significance of comprehending how demographic factors affect diagnostic proficiency.^[8]^

Despite these results, there is still a significant research gap that focuses on the relationship between ECG interpretation skills across a range of demographics and cognitive engagement as assessed by cutting-edge techniques like eye-tracking technology. Few studies have examined the underlying cognitive processes that contribute to these differences, despite the fact that prior research has concentrated on identifying differences in diagnostic accuracy.^[9]^ To close this gap, this study used eye-tracking technology to learn more about how clinicians from different demographics interact with ECG data. This information will then be used to develop training programs that are specifically tailored to improve ECG interpretation abilities for all groups.^[10]^ Several studies have investigated training and experience on the skill in interpreting ECGs, as shown in Table 1. One study, for example, showed that fellows in cardiology had better diagnostic accuracy than their counterparts in noncardiology, highlighting the importance of specialized training in building strong ECG interpretation skills.^[11]^ Comparative studies have also shown that healthcare providers who underwent specialized ECG training were more likely to identify a range of cardiac disorders.^[12]^ These results imply that targeted educational initiatives can improve the diagnostic skills of medical professionals.^[13]^

**Table 1:** Demographics of participants categorized by expertise. A total of 72 participants was included in the study.

Feature	Categories
Expertise category	Medical students (junior year): n = 5 Medical students (senior year): n = 11 Resident: n = 1 Fellows: n = 10 Technicians: n = 10 Cardiac care unit nurses: n = 5 Catheterization lab nurses: n = 6 General nurses: n = 4 General doctors (not cardiology specialists): n = 2 Consultants: n = 9
Age	20–23 yr: n = 10 23–25 yr: n = 9 26–30 yr: n = 21 30–35 yr: n = 11 35–45 yr: n = 12
Gender	Male: n = 51 Female: n = 12
Years of experience	0 yr: n = 10 1 yr: n = 9 2–5 yr: n = 15 5–10 yr: n = 22 15+ yr: n = 7

A total of 72 participants were included in the study.

Definitions of expertise categories:

• Junior medical students: preclinical curriculum.

• Senior medical students: clinical curriculum.

• Nurses: either serving in the catheterization laboratory or cardiac care unit.

• Technicians: cardiovascular technologists in the cardiac catheterization laboratory.

• Fellows: physicians undergoing postgraduate training in cardiology.

• Consultants: board-certified independent cardiology practitioners.

• Residents: medical doctors in the cardiology residency program.

According to one study, younger medical professionals – especially those who have just completed medical school – tended to outperform more experienced professionals in ECG recognition tasks.^[14]^ Increased exposure to modern educational technologies and methodologies during training may be the cause of this discrepancy.^[15]^ Furthermore, older doctors who may have relied on conventional learning methods may find it difficult to use newer diagnostic techniques, which could negatively impact their performance.^[16]^ Studies have indicated that male practitioners frequently perform better than their female counterparts in ECG recognition tasks, and gender disparities in medical education have also been documented. For example, a study highlighted that male medical students displayed higher ECG interpretation accuracy compared to female students, potentially because of differences in educational exposure and confidence levels.^[17]^ This would help develop appropriate training programs to equip all health professionals equally.

Despite the growing number of studies that has investigated ECG interpretation competence, there is still a notable gap in the literature on the underlying cognitive processes that determine such competencies. Many previous studies have focused primarily on recognition accuracy and neglected practitioners’ cognitive engagement during ECG analysis.^[18]^ This gap can be addressed well by using innovative methodologies, such as eye-tracking metrics, to ascertain how healthcare providers interact with ECG data and reveal cognitive load and detailed processes of decision-making involved in interpretation.^[19]^ The analysis of eye movements would help researchers identify areas of difficulty that practitioners encounter, and thus inform targeted educational strategies.^[20,21]^

This calls for a study on different cadres of medical professionals’ proficiency in identifying ECG abnormalities, including diverse categories of expertise level, gender, age, and years of experience. This study helped gain an in-depth understanding of ECG interpretation skills that exist in clinical practice by recruiting a diverse cohort of 72 participants comprised of medical students, residents, and cardiology consultants. The investigation also analyzed multiple ECG abnormalities and used eye-tracking methodologies to assess cognitive engagement during interpretation tasks.^[22]^

Recent studies have shown that eye-tracking technology can enhance our understanding of cognitive engagement in medical tasks, such as ECG interpretation, significantly by providing insights into visual attention patterns and decision-making processes.^[20,21]^ This innovative method allows the specific challenges that healthcare professionals face to be identified, thereby informing targeted training interventions that can improve diagnostic skills and patient care outcomes.^[17,22]^

The findings have important implications for medical education and clinical practice. By identifying the gaps in ECG recognition skills across various professional categories, the study’s findings could improve the diagnostic capacity of health professionals and aid in the development of focused educational initiatives. Enhancing ECG interpretation skills should ultimately result in better patient care outcomes, which highlights the significance of specialized educational approaches that take into account the different training and experience levels of healthcare professionals. To improve diagnostic quality and guarantee consistent patient care, it is essential to comprehend the variation in ECG interpretation abilities among healthcare professionals. To standardize ECG interpretation across various practitioner groups, customized training programs can be created by identifying specific gaps in knowledge, age, and gender. Reducing this variability not only supports diagnostic accuracy but also promotes timely, effective interventions that lead ultimately to improved patient outcomes and a higher standard of care

## 2. Methods

This study was designed to evaluate various medical professionals’ ability to recognize ECG abnormalities and assess their performance across different categories.

### 2.1. Participants

A total of 72 subjects was recruited, including junior and senior medical students, cardiology consultants, residents, fellows, technicians, and nurses. The participants were categorized further by gender, age, and years of experience. Such a varied group allowed recognition to be evaluated well according to ECG skills at various levels of experience and skill. By including various roles, the study analyzed how different professionals perform in recognizing ECG abnormalities.

### 2.2. Exposure to ECG abnormalities

Participants were exposed to different ECG abnormalities, including normal sinus rhythm (NSR), atrial fibrillation (AFib), ventricular tachycardia (VT), Wolff–Parkinson–White (WPW) syndrome, and left bundle branch block (LBBB). By exposing the participants to frequent and complex ECG conditions, their diagnostic ability was put to a broad test. Understanding the way that professionals identify a range of abnormalities can highlight areas for improvement in training and education.^[23,24]^

### 2.3. Data collection

The study employed a series of short and long ECG segments, and both “static areas of interest” (AOI) and “stimulus” events were captured and analyzed. Using both short and long ECG segments allowed the way that participants engage with different types of data presentations to be compared. Short segments offer a snapshot of specific abnormalities, while longer segments provide a continuous view that may reflect real-life scenarios in clinical settings better. This dual approach helped assess different aspects of ECG interpretation skills.

### 2.4. Analysis

The data for this analysis were drawn from 2 key references.^[23,24]^ Several figures and tables were constructed that allowed the participants’ performance in detecting ECG abnormalities to be analyzed and visualized. Visualization techniques show the presence of trends and patterns in data and, therefore, make it easier to interpret complex information. This approach provides a better and clearer understanding of participants’ performance that is helpful in the presentation of findings.

### 2.5. Eye-tracking metrics

Metrics included “first fixation duration,” “average fixation duration (AFD),” “time spent,” “fixation count,” and mouse clicks. These were analyzed across both short and long ECG segments. Eye-tracking metrics such as these provide insight into participants’ engagement and cognitive processes while they are interpreting ECGs. Understanding where and how long participants focus can highlight areas of difficulty and inform targeted training interventions.

### 2.6. Eye-tracking implementation

Eye-tracking technology was used to monitor and record participants’ visual attention as they interpreted ECG readings. The participants were equipped with an eye-tracking device that recorded their Gaze data by capturing fixation duration, saccade patterns, and AOIs on the ECG displays. Specific metrics, such as total fixation time, AFD, and the number of fixations were calculated to evaluate how long and how often participants focused on different segments of the ECG. These data provided insights into their cognitive engagement and areas where they encountered difficulties.

### 2.7. Machine learning (ML) implementation

This study employed a Random Forest classifier to predict ECG abnormalities classified as “short” and “long.” The dataset was prepared by extracting relevant features from ECG readings with each instance labeled accordingly. The model was trained using a subset of the data, and k-fold cross-validation was applied to ensure the ability to generalize the results and reduce the risk of overfitting. Performance was evaluated using such metrics as accuracy, precision, recall, and F1-score, as well as a confusion matrix that provided detailed insights into the predictions. Feature importance analysis was also conducted to identify which ECG characteristics had the most influence on the classification outcomes. These insights offer potential for targeted training programs intended to improve healthcare professionals’ ECG interpretation skills. This methodology ensured a comprehensive and reliable approach to ECG classification, with opportunities for future enhancements through hyperparameter tuning and exploration of other ML algorithms.

### 2.8. Ethical declaration

The Institutional Review Board (IRB) of the Qatar Biomedical Research Institute at Hamad bin Khalifa University approved the study and data collection (research protocol number QBRI-IRB-2020-01-009) before the experiment commenced. This guaranteed that the IRB approval followed all of the study methods according to international regulatory agencies’ guidelines and recommendations. Written informed consent was obtained from all participants in this study.

### 2.9. Definitions of expertise categories

- Junior medical students: preclinical curriculum
- Senior medical students: clinical curriculum
- Nurses: either serving in the catheterization laboratory or cardiac care unit
- Technicians: cardiovascular technologists in the cardiac catheterization laboratory
- Fellows: physicians undergoing postgraduate training in cardiology
- Consultants: board-certified independent cardiology practitioners
- Residents: medical doctors in the cardiology residency program

### 2.10. ECG abnormalities recognition

The frequency and distribution of ECG abnormalities that the participants recognized were documented, and further analysis by gender and age group was conducted. Analyzing recognition rates across different demographics allowed trends and disparities in ECG interpretation skills to be identified. This knowledge can inform future training programs tailored to specific needs to ensure that all professionals are equipped adequately to recognize ECG abnormalities.

Demographic data for the 72 participants in this study classified by professional expertise, age, gender, and years of experience are shown in Table 1. They included a diverse cadre of medical professionals comprised of medical students (junior and senior), residents, fellows, technicians, nurses, general doctors, and consultants. The largest groups were senior medical students, fellows, and technicians, in which the participants ranged in age from 20 to 45 years. The majority of the participants were male (n = 51), and their years of experience varied from none to over 15 years.

Table 2 defines the ECG abnormalities to which the participants in the study were exposed. These ranged from very common conditions, such as NSR, AFib, hyperkalemia, atrial flutter, and VT to more complex conditions, including WPW syndrome, LBBB, and different types of ST-elevation myocardial infarction. Atrioventricular block and complete heart block were also included.

**Table 2:** The ECG abnormalities to which participants were exposed.

ECG abbreviation	Definition
NSR (normal sinus rhythm)	A normal electrocardiogram rhythm.
AFib or AF (atrial fibrillation)	Irregular, often rapid heart rate causing poor blood flow.
Hyperkalemia	Potassium level imbalance in the blood (higher than normal).
Atrial flutter	Arrhythmia causing a rapid heart rate.
VT (ventricular tachycardia)	Fast ventricular rate.
WPW (Wolff–Parkinson–White syndrome)	An accessory pathway between the atria and ventricles.
Ventricular paced rhythm	Artificial pacemaker inserted to activate the ventricle(s).
LBBB (left bundle branch block)	Blockage of electrical impulses traveling down the left bundle branch.
STEMI (ST-segment elevation myocardial infarction)	Heart attack with a blocked major artery, with different types including anterior, inferior, lateral, anterolateral, and inferolateral STEMI.
Atrioventricular block (complete heart block)	Nerve impulse in the sinoatrial node cannot propagate to the ventricles.

ECG = electrocardiogram/electrocardiography.

Table 3 outlines the participants’ responses across various ECG conditions categorized by gender and age group. Both males and females responded to each ECG condition, with notable differences in the number of responses across age groups. These responses were categorized further by the number of participants exposed to short ECGs (static areas of interest) and long ECGs (stimulus).

**Table 3:** Demographic breakdown of participants and their responses to various ECG conditions.

Demographic	Normal sinus rhythm (NSR)	Atrial fibrillation (AFib)	Left bundle branch block (LBBB)	Ventricular tachycardia (VT)	Short ECG (static AOI)	Long ECG (stimulus)
Gender						
Female	11	11	11	11	220	110
Male	52	52	52	52	1040	520
Age group						
20	2	2	2	2	40	20
22	1	1	1	1	20	10
30	1	1	1	1	20	10
32	1	1	1	1	20	10
40	1	1	1	1	20	10

ECG = electrocardiogram/electrocardiography.

**Figure 1.**
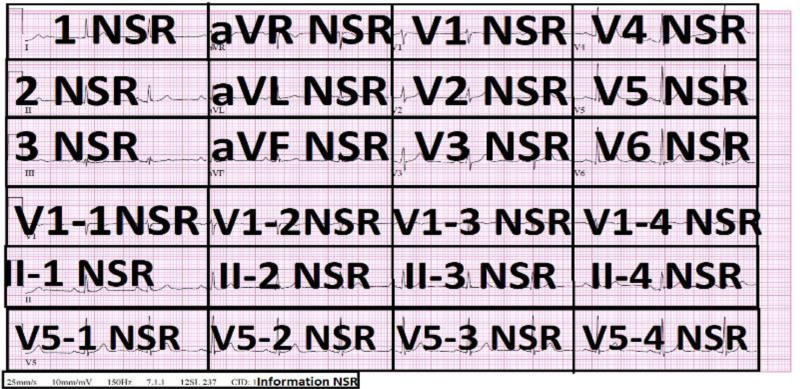
Segmented ECG grid layout with normal sinus rhythm (NSR) annotations. ECG = electrocardiogram/electrocardiography.

Figure 1 shows a 12-lead ECG with each segment labeled “NSR”. The standard limb leads (I, II, and III), augmented limb leads (aVR, aVL, and aVF), and precordial leads (V1–V6) are shown in their respective boxes. The ECG traces exhibited NSR across all leads, with regular P and T waves, and QRS complexes. Each segment is demarcated clearly within each box, with lead labels and rhythm identification marked prominently. The annotation follows the format “Lead NSR” that emphasizes the observation of a normal rhythm throughout the recording. Standard calibration markers of 25 mm/s and 10 mm/mV are noted at the bottom, together with system.

**Figure 2.**
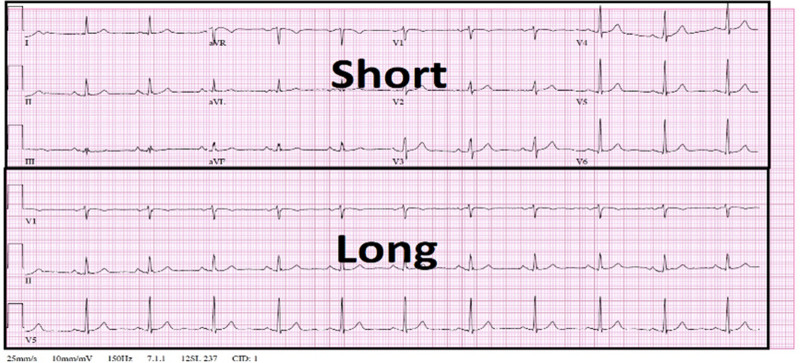
Comparison of short and long ECG segments. ECG = electrocardiogram/electrocardiography.

Figure 2 provides a comparison between short and long ECG segment displays. The top portion labeled “Short” shows typical ECG leads (I, II, III, aVR, aVL, aVF, and V1–V6) in a compact format that allows the heart’s electrical activity to be visualized quickly from various perspectives. The bottom portion labeled “long” shows continuous traces for selected leads (V1, II, and V5) that offers an extended view of the electrical activity to assess the rhythm’s consistency over time better. This format allows clinicians to focus on the regularity of heart rhythms over longer intervals while they can retain the lead-specific details of the short format.

The data gathered from these diverse medical professionals provided insights into the challenges faced in recognizing ECG abnormalities. The analysis focused on short versus long ECG segment performance, eye-tracking metrics, and the application of ML classifiers, all of which contributed to a better understanding of participants’ behaviors and areas for future improvement in ECG interpretation training.

## 3. Results

The 72 participants, including medical students, residents, fellows, technicians, nurses, and consultants, had an average age of 29.47 ± 6.15 years.

**Figure 3.**
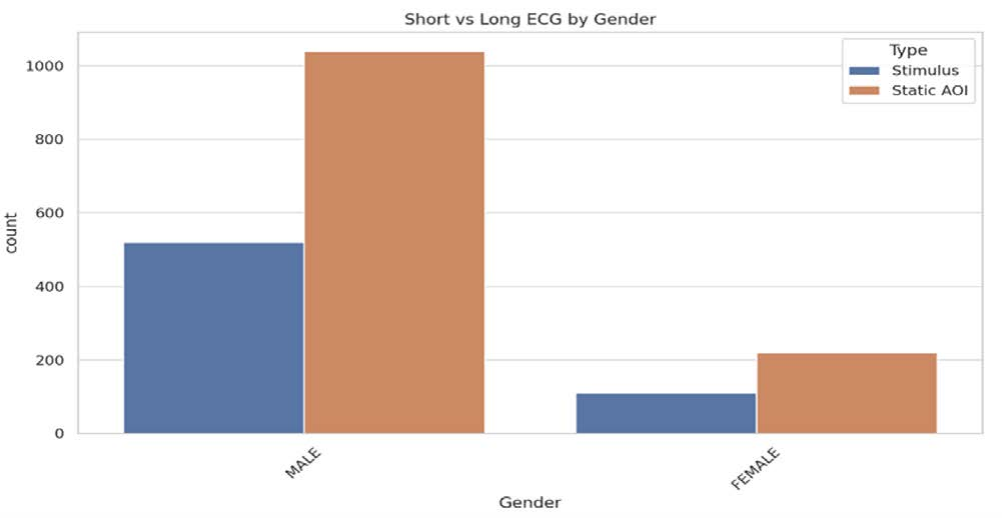
Short versus long ECG by gender. ECG = electrocardiogram/electrocardiography.

Figure 3 shows the distribution of short and long ECG data between males and females. The “stimulus” and “static AOI” categories were compared across gender. The figure shows that males have a notably higher count of both “stimulus” and “static AOI” data, in which “static AOI” was substantially higher. Females have lower counts for both categories overall, although “static AOI” still dominates. The disparity between male and female counts is evident, as males have more than double the number of observations in both categories.

**Figure 4.**
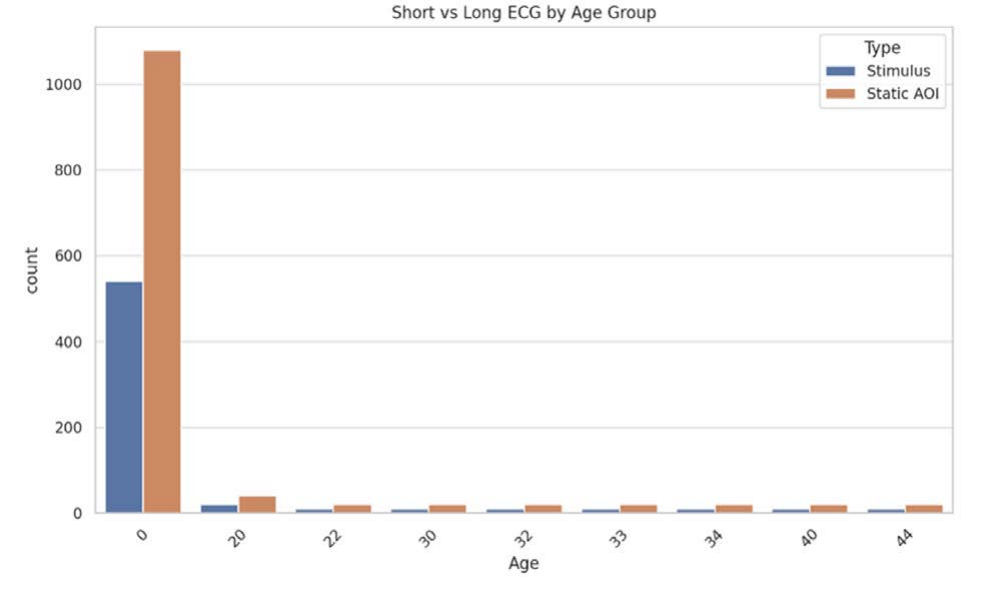
Short versus long ECG by age group. ECG = electrocardiogram/electrocardiography.

Figure 4 illustrates the distribution of short and long ECG data across various age groups. The data are categorized into 2 types: “stimulus” and “static AOI.” The y-axis represents the count of observations, while the x-axis represents different age groups, with a notable concentration in the youngest group (0 years). The younger age group (0 years) shows a significantly higher count of both “stimulus” and “static AOI” data, particularly with a larger portion of “static AOI.” The counts are notably lower across other age groups, with minimal variance between stimulus types.

**Figure 5.**
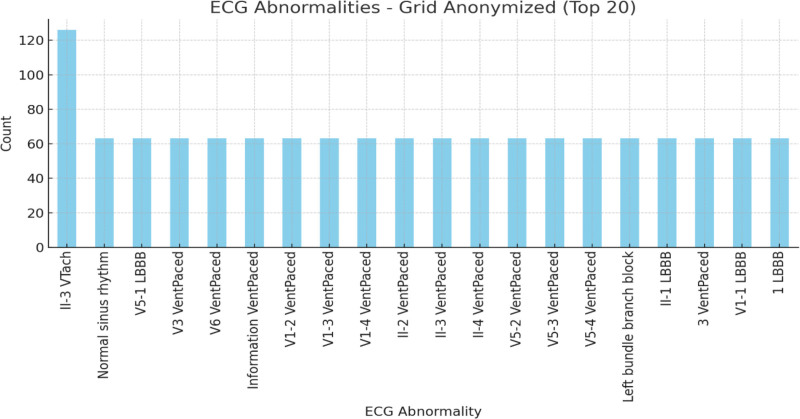
ECG abnormalities–grid-anonymized. ECG = electrocardiogram/electrocardiography.

Figure 5 represents the top 20 most common ECG abnormalities in a grid-anonymized dataset. The x-axis shows various ECG abnormalities, while the y-axis indicates the frequency of occurrence (count). The most frequent abnormality, “II-3 VTach” (ventricular tachycardia), occurred over 120 times, followed by several paced rhythms such as “V1-3 VentPaced,” each with approximately 60 occurrences. Other notable abnormalities include “Normal sinus rhythm,” “V5-1 LBBB”, and different forms of paced rhythms, each of which occurred with similar frequencies.

**Figure 6.**
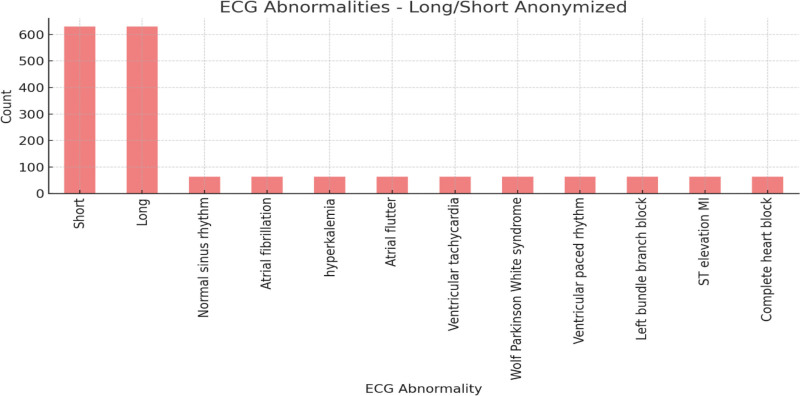
ECG abnormalities–long/short anonymized. ECG = electrocardiogram/electrocardiography.

Figure 6 summarizes the distribution of long and short ECG abnormalities in a long/short anonymized dataset. The x-axis lists ECG abnormalities, while the y-axis shows the frequency of each abnormality. The “short” and “long” categories dominate with more than 600 occurrences each. Other abnormalities, including “NSR,” “AFib,” and “VT,” appear much less frequently, with fewer than 50 occurrences each. This highlights the overwhelming presence of “short” and “long” abnormalities compared to other ECG issues.

**Figure 7.**
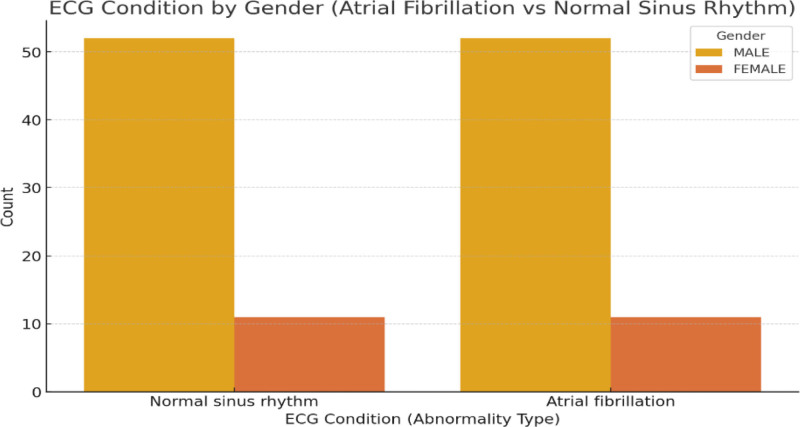
Distribution of ECG conditions by gender (atrial fibrillation vs normal sinus rhythm). ECG = electrocardiogram/electrocardiography.

Figure 7 illustrates the distribution of 2 ECG conditions, AFib, and NSR by gender. The x-axis represents the ECG condition, divided into 2 categories: NSR and AFib. The y-axis indicates the number of individuals within each category.

The figure is broken down further by gender, where male participants are shown in yellow and female participants in orange. It is evident that the majority of individuals with NSR are male, while a smaller number of females are represented. Similarly, in the AFib category, males outnumber females, although the total occurrences for both genders are notably smaller compared to the NSR group. This visualization underscored a higher prevalence of NSR overall across both genders, with a relatively low incidence of AFib.

**Figure 8.**
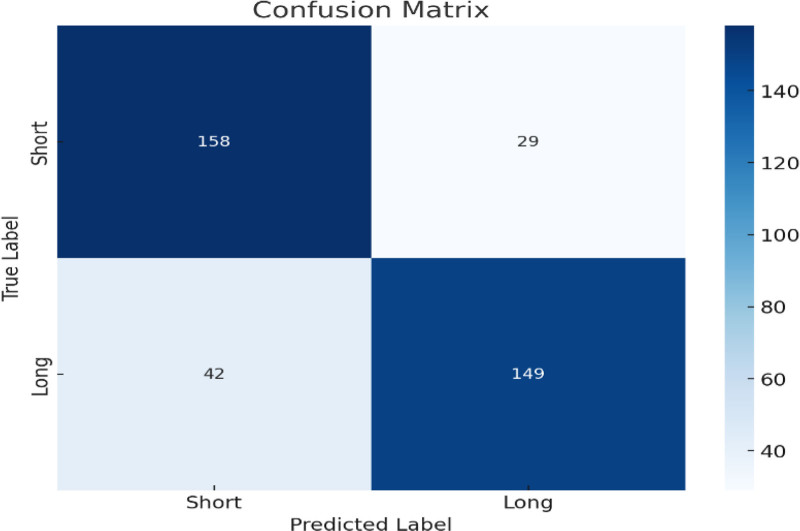
Random Forest classifier for long versus short ECG. ECG = electrocardiogram/electrocardiography.

Figure 8 shows the performance of a Random Forest classifier in predicting ECG abnormalities categorized as “short” and “long.” The true labels are represented on the y-axis, and the predicted labels on the x-axis. The matrix indicates that the model predicted 158 instances of “Short” and 149 instances of “long” correctly. However, there are misclassifications, in which 29 “short” cases were predicted incorrectly as “long” and 42 “long” cases were misclassified as “short.” The color intensity represents the number of predictions, where darker shades indicate higher frequencies. A confusion matrix and feature importance chart show that the Random Forest model achieved 81% accuracy in classifying long versus short ECG readings.

**Figure 9.**
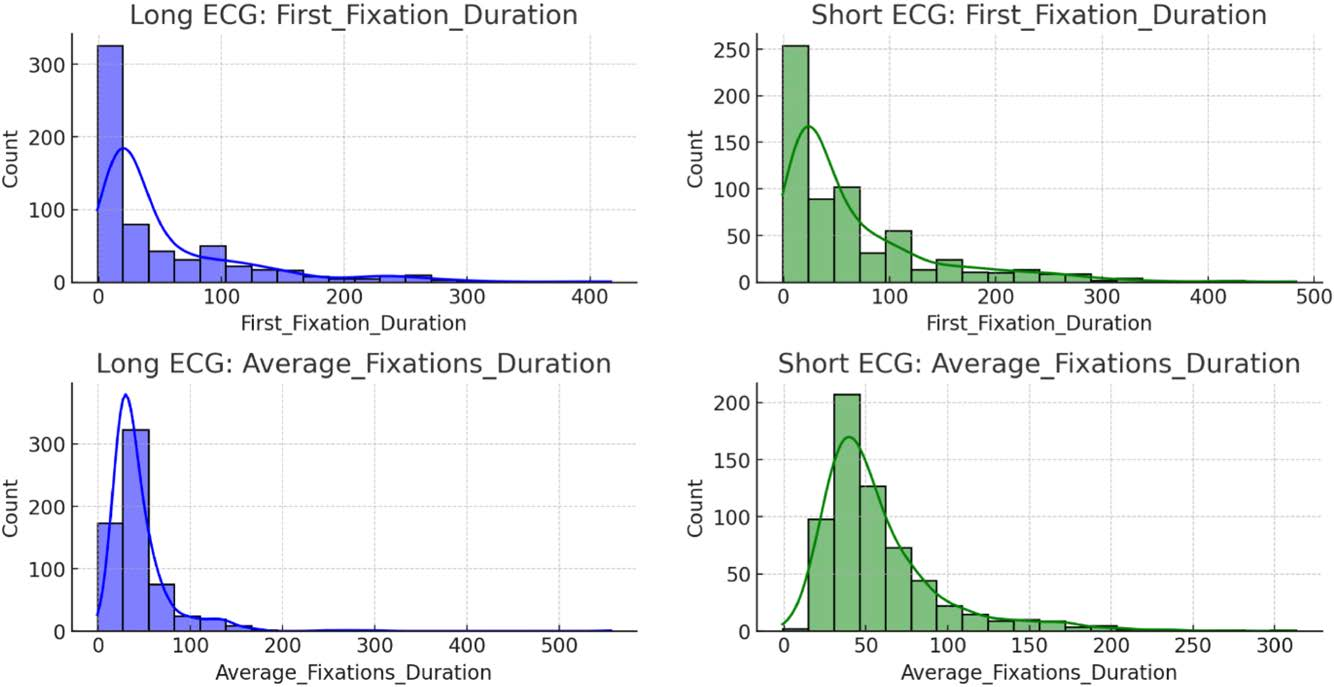
Comparison of “first fixation duration” and “average fixation duration” between long and short ECG events. ECG = electrocardiogram/electrocardiography.

The top-left panel in Figure 9 shows that the “first fixation duration” for long ECG events is concentrated at lower values, largely between 0 and 50 ms, with a long tail extending to higher durations that indicates a right-skewed distribution. In contrast, the top-right panel shows a similar distribution for short ECG events, but with a slightly higher frequency of intermediate fixation durations compared to the long ECG group. The bottom-left panel demonstrates that “AFD” in long ECG events follows a similar right-skewed pattern, in which most of the fixations were concentrated at durations less than 100 ms. In comparison, the bottom-right panel reveals that the “AFD” in short ECG events tends to be centered more around 50 to 100 ms, suggesting a tighter distribution for this metric compared to the more dispersed long ECG fixations.

**Figure 10.**
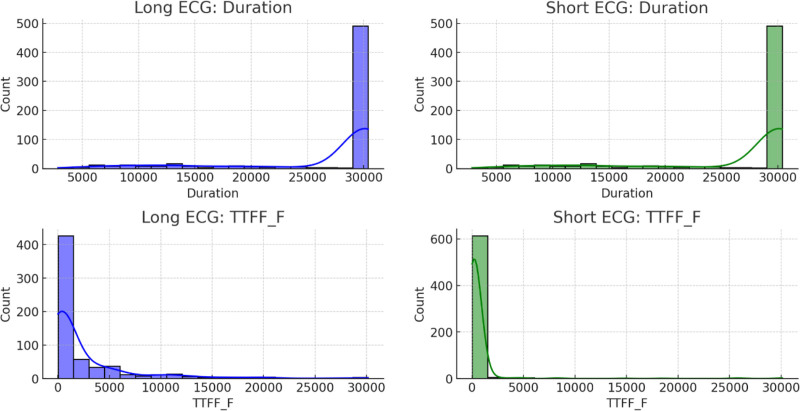
Comparison of the “duration” and “time-to-first-fixation” metrics between long and short ECG events.

In the top-left panel in Figure 10, the distribution of “Duration” for long ECG events is skewed highly to the right, as the majority of the events clusters around the higher end near 30,000 units. A similar pattern is observed in the top-right panel for short ECG events, in which most of the durations also peak at approximately 30,000, although there is generally less variability in the lower ranges. The bottom-left panel displays the distribution of the “time to first fixation – fixated” variable for long ECG events, which is right-skewed sharply, indicating that most events had shorter fixation times, while some extend to much higher values. This pattern is mirrored in the bottom-right panel for short ECG events, in which the “time to first fixation – fixated” distribution also peaks near zero and tapers off rapidly, showing that short fixation times are common in both the long and short ECG groups.

**Figure 11.**
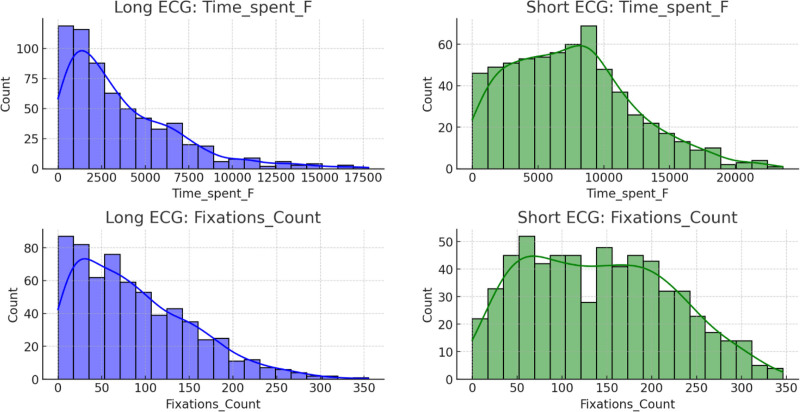
Comparison of “time spent” and “fixation count” between long and short ECG events. ECG = electrocardiogram/electrocardiography.

**Figure 12.**
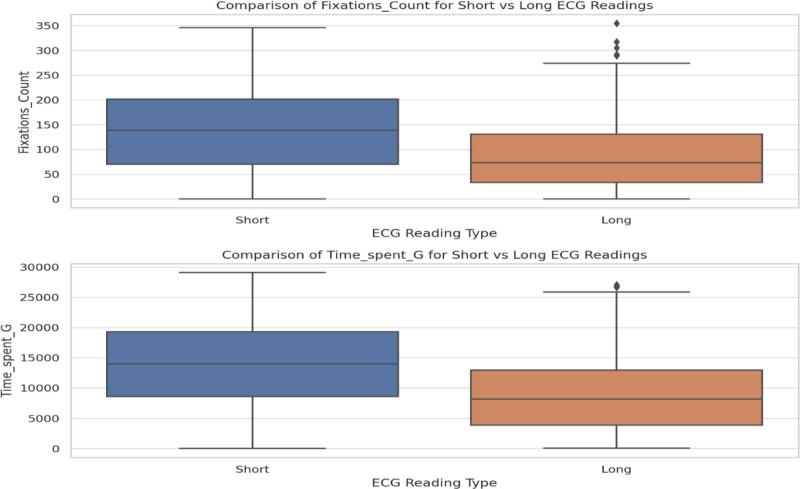
Comparison of time spent G (total time) for short versus long ECG readings. ECG = electrocardiogram/electrocardiography.

**Figure 13.**
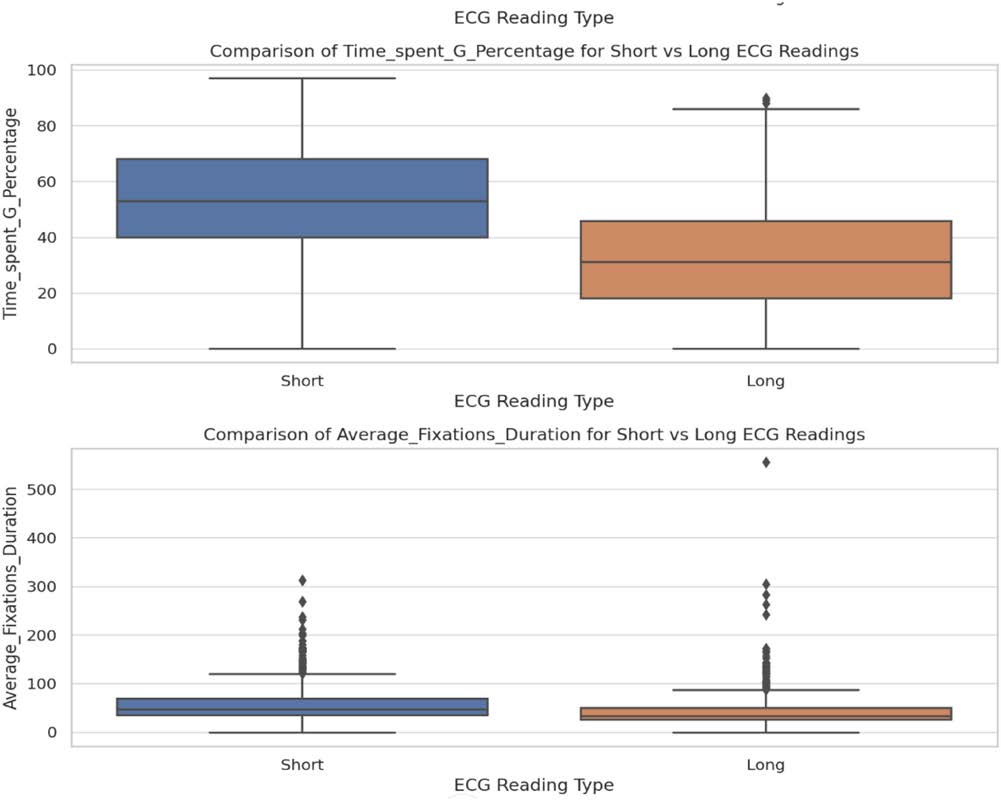
Comparison of fixation count for short versus long ECG readings. ECG = electrocardiogram/electrocardiography.

The top-left panel in Figure 11 shows that “time spent” in long ECG events has a broad distribution that peaks between 2000 and 3000 units and decreases gradually with longer durations. Meanwhile, the top-right panel shows that short ECG events display a similar distribution, but with a relatively higher count for longer durations. The bottom-left panel reveals that “fixation count” for long ECG events follows a left-skewed distribution, in which the majority of the values fall between 0 and 100 fixations, and fewer instances as the fixation count increases. On the other hand, the bottom-right panel shows that short ECG events exhibit a more evenly distributed fixation count, with values spread more across the range of 0 to 200 fixations, indicating greater variation in the number of fixations during short ECG events. In Figure 12, the box plot shows the total time spent on short and long ECG readings. The time spent on short readings tends to be higher, with a median value of approximately 15,000 ms and significant variability, as indicated by the wide interquartile range. Long readings have a lower median time spent (~10,000 ms) and a tighter distribution with some outliers that represent longer engagement. This suggests that users tend to spend more time on shorter readings. The box plot in Figure 13 compares the fixation counts between short and long ECG readings. The box for short readings indicates a median fixation count of approximately 150, with a broader interquartile range, which suggests variability in users’ behavior. For long readings, the box is narrower, with a median of approximately 100, as well as some outliers that indicate much higher fixation counts. Overall, short readings appear to engage users more frequently with respect to fixations, although variability exists.

**Figure 14.**
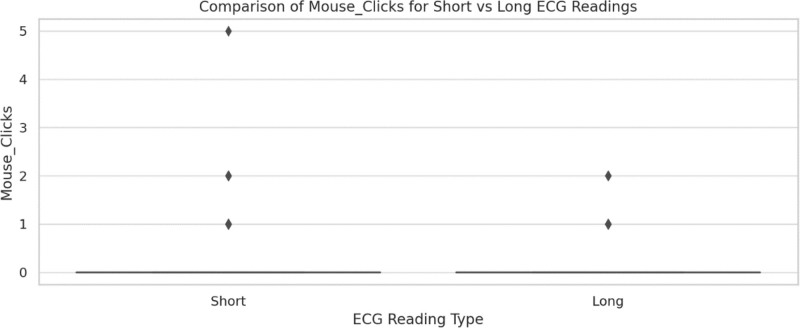
Comparison of mouse clicks for short versus long ECG readings. ECG = electrocardiogram/electrocardiography.

Figure 14 illustrates the number of mouse clicks recorded for short and long ECG readings. The graph displays a scatterplot with data points that represent the distribution of clicks for both reading types. The majority of the values for both short and long readings show minimal clicks, with some higher outliers. The graph suggests that there is little variation between the 2 types of readings in mouse click activity.

## 4. Discussion

This study was conducted to assess various medical professionals’ proficiency in recognizing ECG abnormalities by analyzing performance across categories such as expertise level, gender, age, and years of experience.^[5,25]^ This research, which included 72 participants who ranged from medical students to cardiology consultants, provided a comprehensive perspective on ECG interpretation skills within clinical practice.^[17]^ The sample’s diversity reflected different levels of training and experience, which are crucial in interpreting ECG readings accurately.

The study differed from previous research in several significant ways. Prior studies often targeted specific cohorts, such as cardiology residents or seasoned practitioners, which limited the breadth of perspectives on ECG interpretation.^[12]^ In contrast, our inclusion of a wide array of medical professionals, from novices to experienced cardiologists, provided a more holistic view of the ECG interpretation landscape.

The participants’ demographics were varied; 20.8% of the respondents were medical students, list the others, while 27.8% were cardiology consultants. This inclusive approach was vital to understand the way that varying levels of expertise and training affected diagnostic capabilities and suggested a potential need for tailored educational interventions.^[26]^

Further, previous research has often focused on a narrow range of ECG abnormalities, and overlooked the complexities of diagnosing more uncommon conditions frequently. Our study’s comprehensive evaluation, which included both common (e.g., NSR) and complex disorders (e.g., VT, WPW syndrome), allowed for a nuanced understanding of practitioners’ diagnostic skills.^[11]^ This breadth of evaluation is essential to identify specific educational needs within the healthcare workforce.

The methodological innovations in this study enhanced its value significantly. Many earlier studies have neglected to include cognitive engagement metrics, and emphasized recognition accuracy primarily.^[20,27]^ By incorporating eye-tracking metrics, such as “first fixation duration” and “AFD,” we illuminated the cognitive processes involved in ECG interpretation. Table 2 presents these eye-tracking metrics, which revealed that participants spent significantly more time analyzing longer ECG segments, and indicated potential difficulties in interpretation. This advancement enriched our understanding of how medical professionals approach ECG analysis and allowed us to identify areas where they may struggle, which can encourage more targeted educational interventions.^[21]^

Our findings’ implications for medical education, clinical practice, and future research are substantial. Recognizing gaps in ECG interpretation skills among different professional categories can inform the development of focused educational programs.^[8]^ Tailoring training interventions based upon demographic analysis and specific performance metrics can enhance healthcare providers’ diagnostic capabilities and ultimately improve patient care. The data in Table 3, which presented recognition rates by demographic categories, underscored the necessity for these tailored interventions.

This research can serve as a benchmark to evaluate existing ECG training programs. By understanding various professionals’ performance, educators can adjust curricula to target areas that need improvement to ensure that all practitioners are equipped to recognize ECG abnormalities effectively.^[4]^

Improved recognition of ECG abnormalities can lead to quicker and more accurate diagnoses, and enable timely interventions that enhance patient outcomes.^[13]^ A correlation was found between recognition accuracy and clinical decision-making efficiency, which reinforces the need for medical professionals’ continuous education and assessment to maintain high standards of care.

This study lays the groundwork for future research by pinpointing areas that require further exploration, such as clinical experience’s influence on ECG recognition skills and the role of continuous professional development in sustaining diagnostic competencies. Our findings from ML techniques, such as the 81% accuracy achieved by a Random Forest classifier, suggest these tools’ potential to enhance ECG interpretation.^[28,29]^ Figure 3 presented performance metrics for the Random Forest classifier and compared its accuracy to traditional methods visually, which thus paves the way for the development of automated diagnostic tools that can help clinicians make more accurate and efficient assessments.

The analysis of the eye-tracking data offered insights into cognitive load’s effect on ECG interpretation. The data revealed a relation between “fixation count” and recognition accuracy, indicating that participants with higher fixation counts tended to have lower recognition rates, which suggests potential challenges in processing complex ECG readings effectively.^[30]^ By identifying such patterns, educators can devise training techniques that focus on improving cognitive strategies with which to interpret ECGs, which will enhance diagnostic performance overall thereby.

Furthermore, variations were found among different age groups’ performance in recognizing ECG abnormalities, and revealed that younger participants (ages 26–30) excelled in identifying common ECG patterns compared to older groups. This finding may reflect the influence of contemporary training practices and the incorporation of advanced technologies in medical education, to which younger practitioners might be more accustomed. Recognizing these age-related differences can inform the development of adaptive learning strategies that cater to all age groups’ specific needs.

This finding might be a result of modern training methods and the use of cutting-edge technologies in medical education, which younger practitioners may be more used to. Understanding these age-related variations can help create adaptive learning strategies that meet the unique requirements of all age groups. Male participants consistently outperformed female participants in identifying ECG abnormalities under a variety of conditions, indicating significant performance differences between the sexes. Investigating the causes of these discrepancies, including variations in educational exposure, confidence levels, and possible biases in training, is necessary in light of this finding. Medical education programs can foster a more equal learning environment that empowers all practitioners, regardless of gender, by addressing these issues.

Participants who received focused educational interventions had their ECG recognition abilities analysed both before and after training. The findings showed a notable increase in accuracy after training, confirming the efficacy of these programs and bolstering the case for continued creation of specialized training materials to fill in the identified knowledge and skill gaps. They also highlight the importance of ongoing education in upholding high standards for clinical practice. ECG readings were evaluated using a confusion matrix produced by the Random Forest classifier. This matrix detailed the classifier’s performance in differentiating between various ECG conditions and highlighted areas of high accuracy and those that need improvement. Analyzing misclassifications emphasized the importance of refining both ML models and training programs for healthcare professionals. Identifying specific ECG patterns that are misclassified frequently can guide future training efforts and ensure focused education on the most challenging aspects of ECG interpretation.

Longitudinal changes in ECG recognition skills among participants were measured over a 6-month training period. Accuracy rates consistently increased, according to the results, especially for complicated conditions like VT and WPW syndrome. This trend highlights the value of consistent training and exposure to various ECG scenarios. It also highlights the necessity of ongoing education programs that enable medical professionals to hone their abilities and adjust to changing clinical challenges. The highest recognition rates across all ECG abnormalities were attained by attending physicians, closely followed by residents, according to a comparison of ECG recognition performance based on participants’ educational backgrounds. Medical students, on the other hand, performed the worst. This result emphasizes how important advanced training and clinical experience are for improving diagnostic abilities. This gap could be closed and medical students better prepared for clinical practice in the future with targeted educational interventions.

Performance differences in ECG recognition between participants who engaged in collaborative learning sessions versus those who studied independently were assessed. The data revealed that collaborative learners demonstrated higher accuracy rates and quicker recognition times. This implies that understanding and retention of ECG interpretation skills can be improved through peer discussions and shared learning experiences. Medical professionals may benefit from a more encouraging and productive learning environment if collaborative learning is incorporated into training programs. In clinical settings, there was a correlation between ECG recognition skills and patient outcomes, showing that healthcare providers who were more accurate in identifying critical ECG abnormalities reported better patient outcomes, such as shorter hospital stays and lower readmission rates. This correlation emphasizes how important accurate ECG interpretation is in the real world and how it affects the standard of patient care. The study reaffirmed the significance of giving ECG training top priority in medical education to improve patient health by emphasising this correlation.

A comparison was made between the effects of various training modalities (such as online courses, practical workshops, and peer discussions) on ECG recognition abilities. According to the findings, participants who took part in practical workshops showed the biggest increase in recognition accuracy, with peer discussion participants coming in second. This research highlights the necessity of an interdisciplinary approach to education that incorporates a range of instructional strategies to meet the needs of students with different learning preferences and styles. Table 4 compares previous studies with the approach proposed.

**Table 4:** Comparative analysis of previous studies and current study on ECG interpretation skills.

Criteria	Previous studies	Current study
Sample population	Limited to specific groups (e.g., medical students, residents) with smaller sample sizes.^[1,6]^	Diverse cohort of 72 healthcare professionals, including students, residents, nurses, and consultants.
Factors analyzed	Focused on demographics (age, gender, experience) without detailed cognitive analysis.^[3,14]^	Examines demographics and cognitive metrics using eye-tracking to assess interpretation challenges.
Use of technology	Rarely used advanced tools; focused on manual interpretations and accuracy comparisons.^[9]^	Used eye-tracking and machine learning (Random Forest) to analyze behavior and improve accuracy.
Types of ECG conditions	Limited to basic conditions (e.g., sinus rhythms, arrhythmias).^[15]^	Covered a broad range of abnormalities, from NSR to complex cases like WPW and STEMIs.
Methodological innovation	Focused on accuracy without addressing cognitive load.^[19]^	Tracked cognitive load through metrics such as fixation duration to identify areas needing training.
Findings on demographic disparities	Identified some variation in accuracy by age, gender, and experience, with limited cognitive insight.^[8,18]^	Found significant skill differences by age, gender, and expertise, supported by eye-tracking data.
Implications for training	Suggested specialized training, but lacked actionable methods.^[26]^	Recommended targeted programs based upon gaps, using eye-tracking and ML to enhance skills.
Future directions	Suggested larger studies and demographic-based programs, lacked long-term focus.^[29]^	Advocated for longitudinal studies and further refinement of ML models to address clinical challenges.

ECG = electrocardiogram/electrocardiography, ML = machine learning, NSR = normal sinus rhythm, STEMI = ST-segment elevation myocardial infarction, WPW = Wolff–Parkinson–White syndrome.

This study’s significance lies in its thorough examination of various medical professionals’ ECG interpretation skills, and the factors that are critical to improvements in education and clinical practice. The findings have several key implications. By including a diverse cohort of participants, from medical students to experienced cardiologists, the study provided a richer understanding of the way that expertise influences ECG recognition skills. This diversity reflects the real-world clinical landscape and underscores the necessity of targeted educational strategies that accommodate varying levels of training. The performance variations found that were based upon educational backgrounds emphasizes the importance of tailored training for medical students to enhance their diagnostic competencies early in their careers.

The analysis of recognition rates across different ECG abnormalities facilitated the identification of specific areas where medical professionals may struggle. By addressing these gaps through focused educational programs, the study promoted the development of targeted training interventions. Longitudinal improvement in ECG skills following specific training was found that reinforces the need for continuous education to maintain and enhance proficiency over time.

The correlation established between ECG interpretation skills and patient outcomes emphasized the critical role that accurate ECG recognition plays in effective clinical decision-making. Improving these skills can lead to better patient care, which highlights training’s direct effect on health outcomes. This relation encourages healthcare institutions to invest in robust ECG training programs to ensure that clinicians are well-equipped to respond to ECG abnormalities promptly.

The findings related to collaborative learning advocate for incorporating peer discussions and teamwork into ECG training programs. Collaborative learning has been shown to enhance engagement and knowledge retention, which makes it a valuable approach in medical education. The results of this study suggested that integrating such strategies could improve healthcare professionals’ diagnostic skills.

ML algorithms trained on extensive datasets of ECG readings and associated clinical outcomes can serve as valuable adjuncts to traditional diagnostic practices. By analyzing vast amounts of data, these tools can identify subtle patterns and anomalies that human observers may overlook. For instance, ML models have demonstrated the ability to detect specific ECG abnormalities, such as arrhythmias or ischemic changes, with high sensitivity and specificity.^[31,32]^ This capability allows clinicians to receive real-time support in their decision-making processes, which ultimately leads to more accurate and timely diagnoses.

Moreover, ML can integrate seamlessly with electronic health records and other clinical systems and provide clinicians with context-aware recommendations based upon patient history and current ECG data.^[29]^ This integration not only streamlines workflow, but also enhances the clinician’s ability to make informed decisions, particularly in high-pressure situations where time is of the essence.

In addition, these tools can be designed to learn from clinician feedback, which improves their accuracy and relevance over time continuously. As clinicians interact with these assistive technologies, their input can be used to refine the models and ensure that they remain consistent with the evolving practices in cardiology and other relevant fields.^[33]^

Further, implementing ML tools in training programs for healthcare professionals can foster a more profound understanding of ECG interpretation. By providing students with access to these advanced technologies, they can develop their skills in interpreting ECGs because they benefit from the support and insights that the ML models generate. This dual approach not only enhances individual clinician competencies, but also contributes to a culture of continuous learning and improvement in diagnostic practices.^[34]^

The disparities in ECG interpretation proficiency observed across different demographic groups may be understood better through the lens of cognitive load theory. This theory posits that learners have a limited ability to process information, which can be affected by the complexity of the task at hand and their prior knowledge.^[30]^ Recent educational approaches that make use of technology and interactive learning environments, for example, may help younger clinicians better manage cognitive load. In contrast, even with their experience, older clinicians may have a higher cognitive load when confronted with new technologies or intricate ECG patterns, which could result in less accurate interpretations. This implies that to improve learning outcomes, training programs should take into account the cognitive load that various demographic groups encounter and modify their instructional strategies accordingly.

Furthermore, the use of constructivist learning theories can aid in the explanation of the variations in ECG interpretation performance across a range of demographics. According to constructivism, learning is an active, contextualized process where people create new knowledge from their interactions and experiences.^[35]^ This is especially important when it comes to ECG interpretation, where exposure to a variety of cases and clinical practice are essential components of experiential learning. Age and gender are 2 examples of demographic variables that may have an impact on the kinds of experiences people have, which may then have an impact on their capacity to develop their knowledge and abilities in ECG interpretation. Different levels of proficiency could be caused, for example, by gender disparities in mentorship or educational opportunities. Therefore, constructivist methods that enable students to participate in group learning activities that draw from their individual backgrounds and viewpoints should be incorporated into educational interventions intended to improve ECG interpretation abilities. The sample size is one of the main drawbacks of this investigation. The results may not be as broadly applicable due to the comparatively small number of participants. In addition, because the sample might not accurately reflect the varied backgrounds, experiences, and training environments of clinicians in other settings, the single-center design of the study may introduce biases. Because these limitations may impact the findings’ applicability to various demographics and healthcare contexts, they should be taken into account when interpreting the results. The self-selection of participants is another possible source of bias. Participants might have been more self-assured or already interested in honing their ECG interpretation skills. The results may be skewed, and the observed relationships between performance and demographic factors may be impacted if this self-selection results in an overrepresentation of people who are already predisposed to excel in ECG interpretation.

Future research directions are necessary to validate and build upon the findings of this study in light of these limitations. Conducting longitudinal studies to evaluate the retention of ECG interpretation skills after training is one promising approach. These studies could determine any factors that contribute to long-term proficiency and assess how well clinicians retain their skills over time. Furthermore, controlled studies on educational interventions that use tools for cognitive engagement, like feedback based on eye tracking, ought to be investigated. These experiments can yield important information about how well cutting-edge training methods improve ECG interpretation abilities. Such studies could compare traditional training methods with those that use advanced technologies to measure cognitive engagement and provide real-time feedback, and thus ultimately validate these tools’ effect on learning outcomes.

By addressing the limitations of this study and exploring these future research directions, the field can develop more robust educational strategies that enhance diverse healthcare professionals’ ECG interpretation skills effectively. This continued research will not only contribute to individual clinician development but also improve overall patient care and safety.

## 5. Conclusion

In summary, this study revealed important differences by gender, age, and level of experience, as well as important insights into the difficulties faced by medical professionals in identifying ECG abnormalities. These results highlight how urgently customized training programs that target particular knowledge and skill gaps across various demographic groups are needed. Continuous education and training are crucial as cardiology develops to guarantee that all medical personnel are prepared to identify and handle ECG abnormalities.

There are significant ramifications for training programs, which should be created to meet the individual learning requirements of different clinicians to improve their ability to interpret ECGs. To make training interesting and efficient, scenario-based training modules that mimic real-life ECG problems can be used to give students the chance to apply what they have learnt in a real-world setting where the cognitive load is minimized. Furthermore, creating peer-learning environments can promote cooperation and information exchange between medical professionals with varying degrees of experience, which will improve their comprehension and abilities even more.

Since timely and appropriate interventions depend on accurate ECG interpretation, such focused educational strategies can improve patient outcomes. This study promotes a multimodal approach to education that integrates a variety of learning strategies, such as simulations, hands-on training, and the integration of technological advancements like artificial intelligence and telemedicine, by highlighting the significance of effective ECG interpretation and its direct impact on patient care.^[36]^ Longitudinal studies should be the main focus of future research to evaluate how well these interventions work to enhance diverse healthcare professionals’ ECG interpretation abilities. Healthcare systems can develop a workforce that is not only capable of interpreting ECGs but also adaptable to the changing needs of patient care by continuously improving training programs in light of these findings. Ultimately, enhancing medical professionals’ skills in this critical area will contribute significantly to improved healthcare delivery and patient safety.^[29]^

## Acknowledgments

The author gratefully acknowledges the technical and financial support provided by the Ministry of Education and King Abdulaziz University, DSR, Jeddah, Saudi Arabia.

## Author contributions

**Investigation:** Eyad Talal Attar.

**Methodology:** Eyad Talal Attar.

**Supervision:** Eyad Talal Attar.

**Visualization:** Eyad Talal Attar.

**Writing – original draft:** Eyad Talal Attar.

**Writing – review & editing:** Eyad Talal Attar.
